# Integration of Visual and Olfactory Cues in Host Plant Identification by the Asian Longhorned Beetle, *Anoplophora glabripennis* (Motschulsky) (Coleoptera: Cerambycidae)

**DOI:** 10.1371/journal.pone.0142752

**Published:** 2015-11-10

**Authors:** Fei L.Yv, Xiaoxia Hai, Zhigang Wang, Aihua Yan, Bingxiang Liu, Yongguo Bi

**Affiliations:** 1 College of Forestry, Agricultural University of Hebei, Baoding, Hebei 071000, P. R. China; 2 Key Laboratories for Germplasm Resources of Forest Trees and Forest Protection of Hebei Province, Baoding 071000, P. R. China; Swedish University of Agricultural Sciences, SWEDEN

## Abstract

Some insects use host and mate cues, including odor, color, and shape, to locate and recognize their preferred hosts and mates. Previous research has shown that the Asian longicorn beetle, *Anoplophora glabripennis* (Motschulsky), uses olfactory cues to locate host plants and differentiate them from non-host plants. However, whether *A*. *glabripennis* adults use visual cues or a combination of visual and olfactory cues remains unclear. In this study, we tested the host location and recognition behavior in *A*. *glabripennis*, which infests a number of hardwood species and causes considerable economic losses in North America, Europe and Asia. We determined the relative importance of visual and olfactory cues from *Acer negundo* in host plant location and recognition, as well as in the discrimination of non-host plants (*Sabina chinensis* and *Pinus bungeana*), by female and male *A*. *glabripennis*. Visual and olfactory cues from the host plants (*A*. *negundo*), alone and combined, attracted significantly more females and males than equivalent cues from non-host plants (*S*. *chinensis* and *P*. *bungeana*). Furthermore, the combination of visual and olfactory cues of host plants attracted more adults than either cue alone, and visual cues alone attracted significantly more adults than olfactory cues alone. This finding suggests that adult *A*. *glabripennis* has an innate preference for the visual and/or olfactory cues of its host plants (*A*. *negundo*) over those of the non-host plant and visual cues are initially more important than olfactory cues for orientation; furthermore, this finding also suggests that adults integrate visual and olfactory cues to find their host plants. Our results indicate that different modalities of host plant cues should be considered together to understand fully the communication between host plants and Asian longhorned beetles.

## Introduction

Phytophagous insects discriminate and recognize host plants and mates based on multiple cues, such as odor (olfactory cues), color, size and shape (visual cues) [1–6]. Compared with either visual or olfactory stimuli alone, combined visual and olfactory stimuli may enhance the accuracy of discriminating host from non-host plants and mates from non-mates. Indeed, many insects have been shown to respond synergistically to host plants and mates via visual, olfactory and tactile sensory modes [7–10]. For example, the combination of visual and olfactory cues of host plants was more attractive than either cue alone for foraging-naïve and foraging-experienced bees [6,11,12]. In addition, the white spotted longicorn beetle, *Anoplophora malasiaca*, uses both olfactory and visual cues to locate and recognize mates at medium to short distances [5,13].

The longicorn beetles comprise more than 35,000 species in approximately 4,000 genera, and many species are important pests of forest, plantation, shelter forest and urban trees. Their host plants include hardwoods, coniferous trees, live trees and dying or dead wood [14]. Such highly variable host plants must lead to tremendous variety in the host- and mate-searching behavior and ecology of these pests. For example, contact pheromones are critical in mate location and recognition in some longicorn beetle species (e.g., *Neoclytus mucronatus*, *Megacyllene caryae*, *Megacyllene robiniae* and *Plectrodera scalator*) [15]. Tactile, visual and olfactory cues were used by adult *A*. *glabripennis* to locate and recognize their preferred mates [16–20]. Thus, some longicorn beetles utilize visual, olfactory or other cues, singly or combined, to find and recognize their mates.

In contrast to mate location, the interaction of visual and olfactory cues for host plant location and recognition by longicorn beetles has received very little attention. Olfactory cues used by adult *A*. *glabripennis* to locate and recognize host plants have been described in previous research. For example, adult *A*. *glabripennis* were significantly attracted by volatile organic compounds (VOCs) emitted from maple trees, and *Acer negundo* and *Acer mono* were superior to *Acer truncatum* and *Acer platanoides* for attracting adult *A*. *glabripennis* [21,22]. However, a detailed report concerning the roles of visual cues in the location and recognition of host and non-host plants has still not been published; in addition, the relative importance of visual and olfactory cues for host plant location and discrimination of non-host plants at long distances and close range in adult *A*. *glabripennis* has not been clarified.

In this study, we tested the behavioral responses of female and male *A*. *glabripennis* to host plant (*A*. *negundo*) location and the discrimination of non-host plants (*Sabina chinensis* and *Pinus bungeana*) as well as the relative importance of visual and olfactory cues of host plants in location and recognition behavior at an 80 cm range. In North America, Europe and China, *Acer* is the most commonly infested tree genus, followed by *Aesculus*, *Alnus*, *Betula*, *Salix*, and *Populus* [23–25]. Notably, 70 000 *A*. *negundo* were attacked by *A*. *glabripennis* in a single year (2004) in Harbin, a city in northern China [26]. In addition, Gaag et al. (2014) reported that *A*. *negundo* was the only tree species infested by *A*. *glabripennis*, although many susceptible host plants, including *A*. *mono*, were present in an urban park in Harbin in August 2012 [27]. *A*. *glabripennis* was not reported to forage on twigs or leaves or lay eggs on any tissue of coniferous species such as *S*. *chinensis* or *P*. *bungeana* [23,25,27]. Therefore, we selected *A*. *negundo* as the host plant and *S*. *chinensis* and *P*. *bungeana* as the non-host plants to test the effects of visual and/or olfactory plant cues for host location and recognition by adult *A*. *glabripennis*.

We performed bioassays with *A*. *glabripennis* females and males that were never exposed to *A*. *negundo* or *S*. *chinensis* and *P*. *bungeana* wood before use in our experiments (i) to test the hypothesis that female and male *A*. *glabripennis* have an innate preference for the visual and/or olfactory cues of host plants (*A*. *negundo*) over those of the non-host plants (*S*. *chinensis* and *P*. *bungeana*), (ii) to determine the relative importance of visual and olfactory cues of *A*. *negundo* for host plant location and recognition, and (iii) to compare the attractiveness of either visual or olfactory cues of *A*. *negundo* with the combination of visual and olfactory cues. We hypothesized that female and male *A*. *glabripennis* utilize both visual and olfactory cues, rather than olfactory cues alone [21,22], to find and recognize host plants, and we expected *A*. *glabripennis* to prefer the cues of *A*. *negundo* to those of *S*. *chinensis* and *P*. *bungeana*.

## Materials and Methods

### Insects

Newly emerged female and male *A*. *glabripennis* were obtained from the Agricultural University of Hebei in Baoding, Hebei Province, China, on *Salix matsudana* cv. *Umbraculifera* Rehd and *Salix babylonica*, in early July and late August 2015. The insects were kept in rearing cages (diameter 24 cm, height 22 cm) at a density of approximately 6 individuals (3 females and 3 males) per cage. The temperature was maintained at 27±1°C with 60±10% relative humidity. Gauze netting (nylon, 16–20 mesh) was placed above the cages to prevent escape. The insects were provided water in moist cotton balls placed on the gauze netting and were fed on fresh branches of *S*. *babylonica* which were renewed every 2 days. Adults were starved for approximately 24 hours before use in behavioral experiments. All adults were used 15–25 days after emergence because mating and oviposition normally occur during this period [28–30].

### Plants

*A*. *negundo*, *S*. *chinensis* and *P*. *bungeana* were grown on the western campus of the Agricultural University of Hebei. *A*. *negundo* is a deciduous tree that has green leaves from June to September in Baoding, Hebei, China, and *S*. *chinensis* and *P*. *bungeana* are evergreen coniferous trees. Cut branches with green leaves of *A*. *negundo*, *S*. *chinensis* and *P*. *bungeana* were collected in the western campus of Agricultural University of Hebei, Baoding, China. Cut edges of branches were covered moist cotton balls. New plant material was collected before each 10 trials with single beetles [31–33]. All plant material used in this study comprised branches approximately one year old and 20 cm long with green leaves, but the host and non-host species exhibit substantial differences in leaf shape.

### The behavior bioassay chamber

**Fig 1:**
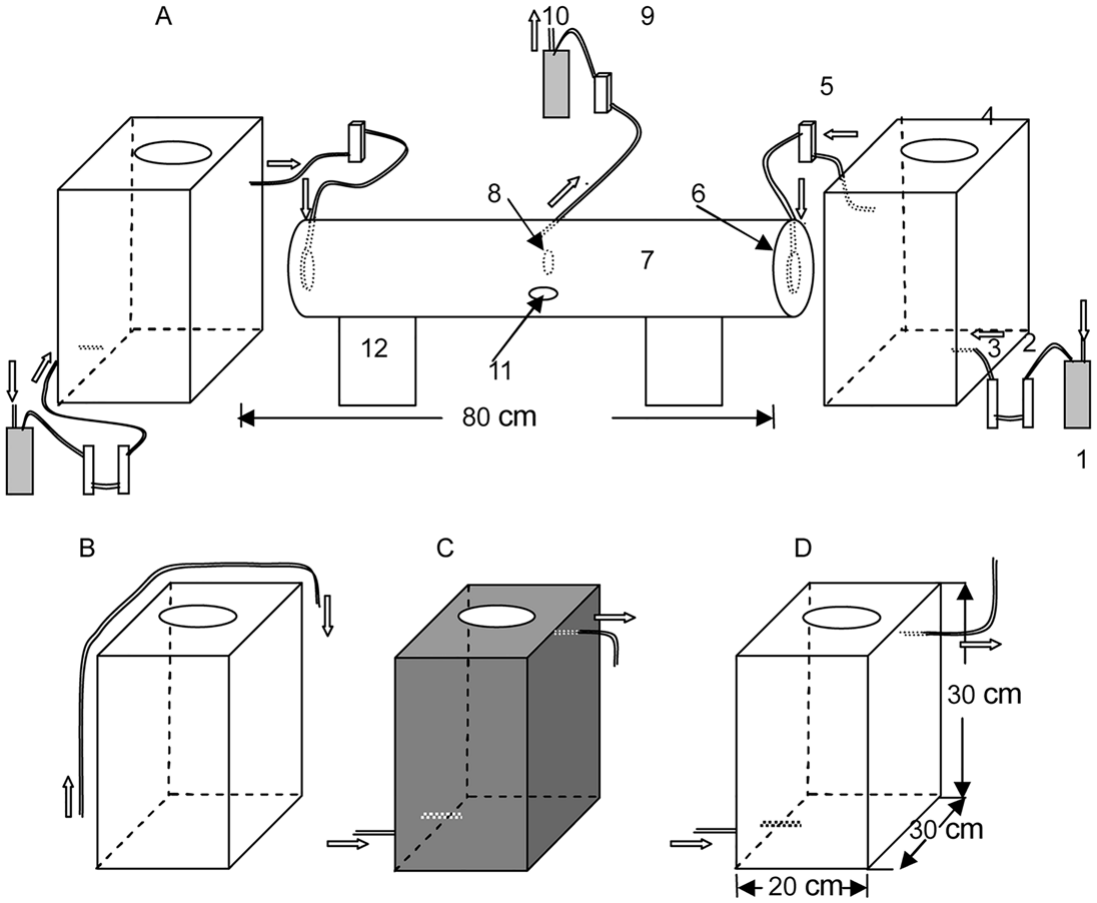
Schematic representation of the behavior bioassay chamber used to test the attraction of *A*. *glabripennis* females and males to visual and/or olfactory cues. (A) host plants and non-host plants cue chambers and test arena; 1, air sampler; 2, glass column with distilled water; 3, glass filter with activated charcoal; 4, one of storage chambers; 5, gas flow meter; 6, air access port of teflon tube; 7, I-tube arena; 8, air exit port of teflon tube; 9, gas flow meter; 10, air sampler, 11, access/exit port of test insects; 12, concave bracing column; the hollow arrows indicate the air flow (300 mL/min) generated with an atmospheric sampling instrument. The storage chambers that provided visual (B), olfactory (C), and visual + olfactory (D) cues of host plants, non-host plants and the blank control for *A*. *glabripennis* females and males.

From early July to the end of August 2015, the responses of adult *A*. *glabripennis* to visual and/or olfactory cues of the host (*A*. *negundo*) and non-host plants (*S*. *chinensis* and *P*. *bungeana*) were studied in the behavior bioassay chamber in the laboratory (Fig 1). The behavior bioassay chamber consisted of an activity I-tube arena that provided space for the activity of *A*. *glabripennis*, two storage chambers into which host or non-host plants were placed to provide visual and/or olfactory cues for *A*. *glabripennis*, a gas supply device, and polytetrafluoroethylene (ptfe; Teflon) tubing (diameter = 8 mm) [11,34]. The activity I-tube arena was 80 cm long×20 cm diameter; the storage chambers were 20 cm long×30 cm high×30 cm wide. The ptfe-tube air entry port in the I-tube arena tail end opened into the I-tube, and the ptfe tube air egress port was made into a circular ring and had 8 holes (approximately 2 mm diameter) opened to the center; and then transparent glass caps were used to prevent air outflow in the two ends of I-tube. Two storage chambers and the activity I-tube were connected together when testing the responses of insects. The system was set up as follows for the various experiments. We used 3 different types of storage chamber as the behavior bioassay chamber to provide the host and non-host plant cues, including visual, olfactory, and combined visual and olfactory cues. 1) For olfactory cues alone (Fig 1C), a layer of black paperboard was placed within the storage chamber to obstruct the visual cues of host and non-host plants. Air containing host or non-host plants’ volatiles from the storage chamber (300 mL/min) was blown out by an atmospheric sampling instrument (Model: QC-2, BMILP Science and Technology Development Co., Ltd., Beijing, China). 2) For visual cues alone, we used a transparent storage chamber (Fig 1B), purified air directly into a gas flow meter and the arena, bypassing the plant odor chamber (300 mL/min). 3) For the combination of visual and olfactory cues, we used a transparent storage chamber (Fig 1D). The plant odors were blown from the storage chamber as described for the olfactory cues alone. After five trials with a single female or male, the I-tube arena was cleaned with water and then with pure ethanol [35,36]. The holes (10 cm diameter) on the top of the storage chamber, through which the host and non-host plants were introduced, were sealed with odorless plastic wrap, and then petroleum jelly and quartz glass (15 cm long×15 cm wide) were added over the plastic wrap to prevent the release of green leaf odor after two branches with green leaves were placed in the storage chambers. In addition, light was provided by two LED lights (White LED: LED12WE27CD, 220–240 V, 50 Hz, Philips Holland, Shenzhen, China), approximately 1500–2000 lx, mounted at equal distances and 40 cm heights above the behavior chamber to provide homogeneous lighting. All tests took place in a windowless laboratory.

### Behavior experiments

Two-choice bioassays were conducted to test whether *A*. *glabripennis* has an innate preference for the visual and olfactory cues (alone and combined) of its host plants over those of non-host plants, as well as to determine the relative importance of single and combined visual and olfactory cues for host plant location at an 80 cm range.

#### Experiment 1: visual and/or olfactory cues of host versus non-host plants

In two-choice bioassays, we offered a choice of host versus non-host plants, with 3 different types of cues (visual, olfactory, visual + olfactory), to *A*. *glabripennis*. Experiment 1.1: (i) visual cues of *A*. *negundo* vs. *S*. *chinensis*, (ii) olfactory cues of *A*. *negundo* vs. *S*. *chinensis*, and (iii) visual + olfactory cues of *A*. *negundo* vs. *S*. *chinensis*. Experiment 1.2: (i) visual cues of *A*. *negundo* vs. *P*. *bungeana*, (ii) olfactory cues of *A*. *negundo* vs. *P*. *bungeana*, and (iii) visual + olfactory cues of *A*. *negundo* vs. *P*. *bungeana*.

#### Experiment 2: the relative importance of visual or olfactory cues of host plants

To determine the behavioral responses of *A*. *glabripennis* to visual and olfactory cues of host plants alone and in combination, we conducted 9 different two-choice bioassays. The two-choice bioassays were performed in the following order:

Experiment 2.0: To remove the visual stimulus of the host plant while retaining the odor stimulus, we used black paper to cover the insides of the storage chamber containing the host plant. To ensure that the black paper did not affect beetle behavior, we conducted the following bioassays: (i) black paper vs. blank control, (ii) white paper vs. blank control, (iii) black paper + host plant odor vs. blank control (transparent box) without plants (no visual stimuli of the plant) + air flow of the host plant odor;

Experiment 2.1: (i) visual cue vs. blank control, (ii) olfactory cue vs. blank control, (iii) visual + olfactory cues vs. blank control;

Experiment 2.2: (i) visual cue vs. olfactory cue, (ii) visual cue vs. visual + olfactory cues, (iii) olfactory cue vs. visual + olfactory cues.

### Bioassay protocol

The behavior bioassays were performed between 9:00 and 18:00, when the *A*. *glabripennis* adults were most active. Each bioassay replicate lasted 10 min or until the beetle made contact with one of the ends of the I-tube chamber and remained there for more than 10 s, and the paired stimuli (visual and/or olfactory cues of host and non-host plants) were arranged 80 cm (the length of the active chamber) apart. The behavioral responses of the female and male *A*. *glabripennis* were recorded as follows: A single test insect was placed in the center of the activity chamber, and the clock was started. (1) The first distal sector towards which the adult orientated (the distance of movement was at least two the length of test insects, approximately 6 cm); (2) The insect was observed for 10 min or until it made contact with one of the ends of the I-tube chamber; if it stayed there for more than 10 s, we recorded that as its first choice (first visit). Insects that had not made a choice within 10 min were designated as non-responders and excluded from analysis. (3) Latency was defined as the time elapsed between release and the moment at which the adult first made contact with one end of the test chamber and remained there for more than 10 s, i.e., between release and making a first choice; (4)The time during which the adult remained in each wall (referred to as permanence).

### Statistical analysis

Preference for one of the two option cues was evaluated by comparing both the numbers of adults that first orientated towards either option and the number of adults that actually visited them. To assess differences in total beetles responses (first orientated and first visits) between the paired treatments in each two-choice bioassay, observed versus expected chi-square tests were performed. Non-responders were recorded but excluded from analysis. We used 2 x 2 contingency tables (based on behavioral responses obtained from bioassays) to test for differences in total beetles responses between female and male, and Fisher’s Exact tests were used as post hoc tests for the 2 x 2 contingency tables. The responses of male and female *A*. *glabripennis* were pooled because no obvious differences were found between the two sexes (Fisher’s exact tests: 0.158<*P*<1.000). The latency to the first visit and the time each adult spent (permanence) in each wall to the first contact was compared between two option cues by means of a two-tailed independent-samples Student t-test or Mann-Whitney test if the heteroscedasticity was severe. All statistical analyses were performed using IBM SPSS Statistics 21.00 for Windows (IBM SPSS Inc., Boston, MA, USA) [37].

## Results

### The first series: the visual and/or olfactory cues of host versus non-host plants

In the first experimental series, adult *A*. *glabripennis* oriented first towards the sector in which contained cues of host plants *A*. *negundo*. When testing combined cues from *A*. *negundo* against those of *S*. *chinensis* and *P*. *bungeana*, adults oriented more frequently first towards the sector that contained combined cues of host plants *A*. *negundo* than towards the other sector that contained those of non-host plants, *S*. *chinensis* and *P*. *bungeana* (Table 1). The difference in first towards of adult *A*. *glabripennis* was significant in the visual or olfactory cues of *A*. *negundo* vs. *S*. *chinensis*, but no significant differences were found in the visual or olfactory cues of *A*. *negundo* vs. *P*. *bungeana* (Table 1).

**Table 1:** Number of first orientations and first visits* by *A*. *glabripennis* in response to host (*A*. *negundo*) and non-host (*S*. *chinensis* and *P*. *bungeana*) plant cues.

Experiment	Type of cue	Options offered to ALB	N		First orientations			First visits		
			Female	Male	No. of first orientating	% response		No. of first visit	% response	
1.1	Visual cues	*A*. *negundo*	21	16	26	100	*X* ^*2*^ = 6.081	26	84	*X* ^*2*^ = 14.226
		*S*. *chinensis*			11		**P = 0.014**	5		**P<0.001**
	Olfactory cues^#^	*A*. *negundo*	22	17	28	100	*X* ^*2*^ = 7.410	30	93	*X* ^*2*^ = 16.000
		*S*. *chinensis*			11		**P = 0.006**	6		**P<0.001**
	Visual +olfactory cues	*A*. *negundo*	21	19	27	100	*X* ^*2*^ = 4.900	31	93	*X* ^*2*^ = 16.892
		*S*. *chinensis*			13		**P = 0.027**	6		**P<0.001**
1.2	Visual cues	*A*. *negundo*	22	19	26	100	*X* ^*2*^ = 2.951	32	95	*X* ^*2*^ = 16.026
		*P*. *bungeana*			15		P = 0.086	7		**P<0.001**
	Olfactory cues	*A*. *negundo*	20	20	24	100	*X* ^*2*^ = 1.600	23	83	*X* ^*2*^ = 5.121
		*P*. *bungeana*			16		P = 0.206	10		**P = 0.024**
	Visual +olfactory cues	*A*. *negundo*	22	20	29	100	*X* ^*2*^ = 6.095	33	93	*X* ^*2*^ = 18.692
		*P*. *bungeana*			13		**P = 0.014**	6		**P<0.001**

*: Preference was assumed when a female or male spent more than 10 s in the one distal wall.

^#^: Olfactory cues indicate the volatile organic compounds of branches of *A*. *negundo*, *S*. *chinensis* or *P*. *bungeana* with green leaves.

The pattern of preference as evaluated by adult *A*. *glabripennis* visitation was similar to that found for orientation (Table 1). Adult *A*. *glabripennis* showed a clear preference in their total responses for cues of their host plant *A*. *negundo* over those of non-host plants. Visual and olfactory cues, alone and in combination, of *A*. *negundo* were significantly more attractive to adults than those of either of the non-host plants, *S*. *chinensis* and *P*. *bungeana* (Table 1).

When testing the attractiveness of the visual and olfactory cues, alone and in combination, of *A*. *negundo* against those of *S*. *chinensis* and *P*. *bungeana*, the latency to the first visit and the time each adult spent (permanence) in each wall to the first contact are shown in S1 Table. Both latency and permanence times did not differ between options.

### The second series: the relative importance of visual and olfactory cues of host plants

In experiment 2.0, black and white paper was used to test the responses of adult *A*. *glabripennis*, we found that white paper were more attractive males than the blank control in the first orientation (Table 2). However, there were no significant differences in attracting adults between black paper and blank control. Similarly, no difference in responses was observed between the black box with holes with odor of *A*. *negundo* vs. the transparent box with holes without plants but with air flow of the plant odor (Table 2). Therefore, a layer of black paperboard was placed within the storage chamber to obstruct the visual cues of host and non-host plants in experiments 2.1 and 2.2. In experiment 2.1, adult *A*. *glabripennis* showed similar response patterns in the two-choice bioassays using only host cues, including visual, olfactory, and visual + olfactory cues. Adults initially orientated more frequently towards the sector that contained visual and olfactory cues, alone and in combination, from host plants (*A*. *negundo*) than towards the other sector (Table 2). In the experiment 2.2, adults orientated more frequently towards the sector that contained a visual or combined cues of *A*. *negundo* than towards the sector with olfactory cues. However, adults showed no preference between the visual + olfactory and visual cues of *A*. *negundo* (Table 2).

**Table 2:** Number of first orientations and first visits* by *A*. *glabripennis* in response to cues from host plant (*A*. *negundo*), black paper and blank control.

Experiment	Type of cue	Options offered to ALB	N		First orientations			First visits		
			Female	Male	No. of first orientating	% response		No. of first choice	% response	
2.0	Visual cues	Black paper	28	25	27	100	*X* ^*2*^ = 0.019	20	87	*X* ^*2*^ = 0.782
		Blank control			26		P = 0.891	26		P = 0.376
		White paper^@^	0	21	15	100	*X* ^*2*^ = 3.857	17	95	*X* ^*2*^ = 9.800
		Blank control			6		**P = 0.0495**	3		**P = 0.002**
	Visual + olfactory cues	Black paper + olfactory cues^§^	20	20	21	100	*X* ^*2*^ = 0.100	19	93	*X* ^*2*^ = 0.027
		Blank control + olfactory cues^§^			19		P = 0.752	18		P = 0.869
2.1	Visual cues	*A*. *negundo*	20	16	24	100	*X* ^*2*^ = 4.000	26	94	*X* ^*2*^ = 9.529
		Blank control			12		**P = 0.046**	8		**P = 0.002**
	Olfactory cues	*A*. *negundo*	21	20	27	100	*X* ^*2*^ = 4.122	29	90	*X* ^*2*^ = 11.919
		Blank control			14		**P = 0.042**	8		**P<0.001**
	Visual +olfactory cues	*A*. *negundo*	19	17	25	100	*X* ^*2*^ = 5.444	29	94	*X* ^*2*^ = 16.941
		Blank control			11		**P = 0.020**	5		**P<0.001**
2.2	*A*. *negundo*	Visual cues	23	39	38	95	*X* ^*2*^ = 4.898	38	90	*X* ^*2*^ = 7.143
		Olfactory cues			21		**P = 0.027**	18		**P = 0.008**
	*A*. *negundo*	Visual cues	24	24	21	100	*X* ^*2*^ = 0.750	13	88	*X* ^*2*^ = 6.095
		Visual +olfactory cues			27		P = 0.387	29		**P = 0.014**
	*A*. *negundo*	Olfactory cues	22	22	11	100	*X* ^*2*^ = 11.000	5	89	*X* ^*2*^ = 21.564
		Visual +olfactory cues			33		**P<0.001**	34		**P<0.001**

*: Preference was assumed when a female or male spent more than 10 s in the one distal wall.

^@^: only males were tested in this experiment.

^§^: olfactory cues indicate the volatile organic compounds of branches of *A*. *negundo* with green leaves.

The pattern of preference as evaluated by adult visitation was similar to that found for orientation (Table 2). We found significant differences between white paper and blank control in experiments 2.0, in experiment 2.1 and 2.2 (Table 2). Again, in experiment 2.0, there were no differences in the frequency of visits between the black paper and the blank control, between the black box with holes with the odor of *A*. *negundo* and the transparent box with holes without plants but with air flow of the plant odor (Table 2).

Similarly, the latency to the first choice and the time each adult spent (permanence) in each wall to the first contact are shown in S2 Table. Males spent significantly more time in the sector that contained a white paper than towards blank control sector in experiment 2.0. But both latency and permanence times did not differ between options in the other experiment.

## Discussion

We found that both female and male *A*. *glabripennis* can discriminate between visual and olfactory cues of host plants and non-host plants and have a clear preference for the visual and olfactory cues of their host plants (*A*. *negundo*) over those of non-host plants (*S*. *chinensis* and *P*. *bungeana*), both separately and in combination. These findings suggest that the mechanisms for host plant location and recognition by *A*. *glabripennis* rely on the interaction of visual and olfactory cues rather than either type of cue alone. Previously, distinct cues of host plants had been considered separately to explain the selection of host plants and the avoidance of non-host plants by *A*. *glabripennis* (see above). Therefore, our findings may reveal new mechanisms by which the interplay between visual and olfactory cues is utilized in host plant location and recognition by some longicorn beetle species.

Adult *A*. *glabripennis* were attracted by visual cues of host plants over those of non-host plants when olfactory cues were removed. In addition, when we offered a combination of visual and olfactory cues of the host plant, adults showed a stronger preference in response to the combined cues than to either cue alone, and adults were more attracted by visual + olfactory cues of host plants than those of non-host plants. These findings also suggested that the *A*. *glabripennis* adults not only depend on olfactory cues [21,22] but also on visual cues when searching for host plants. To date, only a few studies have suggested that longicorn beetles have a clear preference for the visual cues (color, shape, etc.) of their host plants, and a trapping experiment performed in the field indicated that this was also the case for *Monochamus* spp. and *Xylotrechus longitarsis* [38,39]. However, we found that visual cues from host plants also play an important role in host plant location and recognition among both female and male *A*. *glabripennis*. In addition, the previous reports stated that adult mating behavior started with visual stimulation of female adults to the male [16,17]. These findings suggest that visual cues from host plants and mates play an important role in the preference host plant and mate location and recognition of *A*. *glabripennis*.

In our two-choice bioassays, adults oriented more frequently towards the sector that contained visual cues of host plant than towards the sector that contained a host plants’ olfactory cues; suggesting that visual cues are initially more important than olfactory cues for orientation at a range of 80 cm. In fact, volatile plant compounds can be highly generalized throughout both host plants and non-host plants [40]. A complex volatile background provided by natural and anthropogenic resources interferes with odor recognition by herbivorous insects, this phenomenon is often suggested as the reason visual cues from host plants are more effective than olfactory ones for long-range signaling [41,42]. However, we cannot exclude the possibility that olfactory cues from host plants might have a function in orientation, because the first orientation of adult *A*. *glabripennis* was significantly difference to the olfactory cues of *A*. *negundo* vs. *S*. *chinensis*; besides adults oriented more frequently towards the sector that contained olfactory cues of host plants than towards the blank control when visual cues were removed. In addition, it has been reported that the six aldehyde compound blend from female were more attractive to males than solvent controls in the Y-tube olfactometer, as well as male-produced pheromone alone or in different combinations with plant volatiles significantly increased trap catches of females in the field [18,43,44].

Our results showed that adult *A*. *glabripennis* were significantly more attracted to the combined cues than to either cue alone. This finding is the first evidence that multiple cues from host and non-host plants, including at least visual and olfactory cues, can mediate the search for host plants and the discrimination of host from non-host plants by *A*. *glabripennis* adults. There are very few reports of longicorn beetle species using the interaction of visual and olfactory cues to locate and recognize their preferred host plants or mates [5,13,40]. However, our research also supports the hypothesis that the interaction of visual and olfactory cues is used not only by bees, flies and moths [11,12,45,46] but also by some longicorn beetle species, at least including *A*. *glabripennis*, *A*. *malasiaca* [5,13], *X*. *longitarsis*, *Monochamus scutellatus* and *Monochamus clamator* [40], for the location and recognition of host plants or mates.

The Asian longhorned beetle, *A*. *glabripennis*, is an economically important invasive pest that has become established in several places in North America and Europe [23], and efforts to develop effective tools for its survey have not been very successful. Both female and male produced long-range sex pheromones attractants for *A*. *glabripennis* have been reported [18,19,20,43,44,47]. Female *A*. *glabripennis* cuticular hydrocarbon extracts contain five olefins, (*Z*)-9-tricosene, (*Z*)-9-pentacosene, (*Z*)-7-pentacosene, (*Z*)-9-heptacosene, and (*Z*)-7-heptacosene, that elicited copulatory behavior in males in laboratory [19]. In laboratory bioassays, male produce a blend of two functionalized dialkylethers, 4-(*n*-heptyloxy)butanal, 4-(*n*-heptyloxy)butanl-1-ol, and (3*E*, 6*E*)-α-farnesene that elicit GC-EAD responses in females [47,48] and are attractive to females, particularly when used with host odors such as (-)-linalool, *trans*-caryophyllene, and (*Z*)-3-hexen-l-ol [20,44]. Indeed, the male- and female-produced pheromone elicits behavioral responses from females and males in laboratory and field bioassays have demonstrated statistically significant but limited attraction [18,43,44]. In our study, the combined visual and olfactory cues were more attractive than either of the host plant cues alone for adult *A*. *glabripennis*. Perhaps a multi-component trap, designed to use a combination of visual and semiochemicals cues, will provide an extremely needed new tool for survey, detection and control of this important forest pest, in the same way that combination of visual and olfactory cues significantly enhanced trap effectiveness for the West Indian sweetpotato weevil, *Euscepes postfasciatus* (Fairmaire) [49].

Understanding the biology, ecology, and behavior of pest insects is essential to produce high-quality trapping tools that ensure the success of control programs. Our results allow a better understanding of the cues used by adult *A*. *glabripennis* during host searching. Further research should contribute to the identification of the chemical compounds from wounded and intact branches of *A*. *negundo* involved in the present study and distinction of the chemical compounds of host and non-host plants. Moreover, considering our finding that white paper was more attractive to males than the blank control, whether the other colors can attract adults should be explored. Further research is needed to ascertain adult *A*. *glabripennis* responses to variations in the combination of the two sensory modalities, such as changes in color and lure dose.

## Supporting Information

S1 Table Mean latency and mean permanence times (in seconds) of *A*. *glabripennis* in response to each cue of host (*A*. *negundo*) and non-host (*S*. *chinensis* and *P*. *bungeana*) plant. (DOC)

S2 Table Mean latency and mean permanence times (in seconds) of *A*. *glabripennis* in response to each cue of host plant (*A*. *negundo*), black paper and the blank control. (DOC)

S3 Table Relevant data underlying the findings described in the manuscript. (DOC)
