# Correction: Integration of Visual and Olfactory Cues in Host Plant Identification by the Asian Longhorned Beetle, *Anoplophora glabripennis* (Motschulsky) (Coleoptera: Cerambycidae)

**DOI:** 10.1371/journal.pone.0146209

**Published:** 2015-12-29

**Authors:** Fei Lyu, Xiaoxia Hai, Zhigang Wang, Aihua Yan, Bingxiang Liu, Yongguo Bi

The first author’s name is spelled incorrectly. The correct name is: Fei Lyu. The correct citation is: Lyu F, Hai X, Wang Z, Yan A, Liu B, Bi Y (2015) Integration of Visual and Olfactory Cues in Host Plant Identification by the Asian Longhorned Beetle, *Anoplophora glabripennis* (Motschulsky) (Coleoptera: Cerambycidae). PLoS ONE 10(11): e0142752. doi:10.1371/journal.pone.0142752

## References

[1] L.Yv F, Hai X, Wang Z, Yan A, Liu B, Bi Y (2015) Integration of Visual and Olfactory Cues in Host Plant Identification by the Asian Longhorned Beetle, *Anoplophora glabripennis* (Motschulsky) (Coleoptera: Cerambycidae). PLoS ONE 10(11): e0142752 doi: 10.1371/journal.pone.0142752 26556100 10.1371/journal.pone.0142752 PMC4640517

