# Supplementary material for: Integration of Visual and Olfactory Cues in Host Plant Identification by the Asian Longhorned Beetle, Anoplophora glabripennis (Motschulsky) (Coleoptera: Cerambycidae)
Source: PLoS One. 2015 Nov 10;10(11):e0142752. doi: 10.1371/journal.pone.0142752 (PMC4640517; doi:10.1371/journal.pone.0142752)
Supplement: S1 Table — (DOC) [file pone.0142752.s001.doc]

**S1 Table. Mean latency and mean permanence times (in seconds) of *A. glabripennis* in response to each cue of host plants (*A. negundo*) and non-host plants (*S. chinensis* and *P. bungeana***).

|  |  |  |  | Latency | | Permanence | |
| --- | --- | --- | --- | --- | --- | --- | --- |
| Experiment | Type of cue | Options offered to ALB | N | Mean ± SE |  | Mean ± SE |  |
| 1.1 | Visual cues | *A. negundo* | 26 | 180.88±18.35 | t=0.702 | 38.96±4.54 | t=1.482 |
|  |  | *S. chinensis* | 5 | 169.20±44.12 | P=0.801 | 23.20±4.22 | P=0.149 |
|  | Olfactory cues# | *A. negundo* | 30 | 159.27±20.48 | t=0.270 | 39.13±3.47 | t=1.549 |
|  |  | *S. chinensis* | 6 | 173.17±53.75 | P=0.789 | 26.50±5.31 | P=0.131 |
|  | Visual +olfactory cues | *A. negundo* | 31 | 139.10±16.20 | t=0.141 | 47.71±6.90 | t=0.120 |
|  |  | *S. chinensis* | 6 | 133.67±24.39 | P=0.889 | 45.67±15.14 | P=0.905 |
| 1.2 | Visual cues | *A. negundo* | 32 | 160.69±21.13 | t=1.134 | 34.91±3.45 | t=0.573 |
|  |  | *P. bungeana* | 7 | 107.57 ±23.63 | P=0.264 | 26.14±5.87 | P=0.274 |
|  | Olfactory cues | *A. negundo* | 23 | 197.47 ±19.68 | t=0.465 | 35.65±5.52 | t=0.787 |
|  |  | *P. bungeana* | 10 | 178.40±43.26 | P=0.645 | 28.30±6.21 | P=0.438 |
|  | Visual +olfactory cues | *A. negundo* | 33 | 157.24 ±13.96 | t=1.657 | 36.15±5.68 | MW=61.000 |
|  |  | *P. bungeana* | 6 | 99.33±28.02 | P=0.106 | 22.67±6.15 | P=0.139 |

#: Olfactory cues refers to the volatile organic compounds of branches of *A. negundo, S. chinensis* or *P. bungeana* with green leaves.

Statistical analysis: t indicates a two-tailed independent-samples Student t-test, MW indicates a Mann-Whitney test.
