# Supplementary material for: Integration of Visual and Olfactory Cues in Host Plant Identification by the Asian Longhorned Beetle, Anoplophora glabripennis (Motschulsky) (Coleoptera: Cerambycidae)
Source: PLoS One. 2015 Nov 10;10(11):e0142752. doi: 10.1371/journal.pone.0142752 (PMC4640517; doi:10.1371/journal.pone.0142752)
Supplement: S2 Table — (DOC) [file pone.0142752.s002.doc]

**S2 Table. Mean latency and mean permanence times (in seconds) of *A. glabripennis* in response to each cue of host plants (*A. negundo***), black paper and the blank control.

|  |  |  |  | Latency | | Permanence |  |
| --- | --- | --- | --- | --- | --- | --- | --- |
| Experiment | Type of cue | Options offered to ALB | N | Mean ± SE |  | Mean ± SE |  |
| 2.0 | Visual cues | Black paper | 20 | 205.40 ± 30.08 | t=0.199 | 26.25 ± 4.01 | t=0.631 |
|  |  | Blank control | 26 | 212.92 ± 23.68 | P=0.843 | 29.81 ± 3.86 | P=0.531 |
|  |  | White paper@ | 17 | 182.94 ± 34.42 | t=0.446 | 30.41 ± 3.93 | t=2.356 |
|  |  | Blank control | 3 | 226.68 ± 140.99 | P=0.661 | 18.00 ± 3.51 | **P=0.045** |
|  | Visual + olfactory cues | Black paper + olfactory cues § | 19 | 169.74 ± 19.03 | t=0.676 | 28.32 ± 3.31 | t=0.861 |
|  |  | Blank control + olfactory cues§ | 18 | 190.06 ± 23.45 | P=0.503 | 34.06 ± 5.90 | P=0.395 |
| 2.1 | Visual cues | *A. negundo* | 26 | 166.93 ± 22.60 | t=0.686 | 47.28 ± 10.16 | t=1.108 |
|  |  | Blank control | 8 | 133.38 ± 44.42 | P=0.497 | 25.38 ± 5.79 | P=0.275 |
|  | Olfactory cues | *A. negundo* | 29 | 157.73 ± 19.64 | t=1.391 | 32.23 ± 4.50 | t=0.873 |
|  |  | Blank control | 8 | 216.00 ± 40.71 | P=0.174 | 24.50 ± 6.21 | P=0.389 |
|  | Visual +olfactory cues | *A. negundo* | 29 | 202.48 ± 20.16 | t=0.684 | 51.76 ± 9.00 | MW=55.000 |
|  |  | Blank control | 5 | 163.60 ± 74.56 | P=0.499 | 57.80 ± 23.21 | P=0.395 |
| 2.2 | *A. negundo* | Visual cues | 38 | 150.34 ± 0.948 | t=0.066 | 33.79 ± 4.61 | MW=342.000 |
|  |  | Olfactory cues | 18 | 148.33 ± 25.29 | P=0.948 | 34.00 ± 6.51 | P=1.000 |
|  | *A. negundo* | Visual cues | 13 | 154.77 ± 21.45 | t=0.156 | 38.08 ± 4.83 | t=0.738 |
|  |  | Visual +olfactory cues | 29 | 160.79 ± 21.45 | P=0.877 | 33.28 ± 3.77 | P=0.465 |
|  | *A. negundo* | Olfactory cues | 5 | 171.60 ± 65.01 | t=0.038 | 40.40 ± 11.32 | t=0.435 |
|  |  | Visual +olfactory cues | 34 | 173.71 ± 19.39 | P=0.970 | 45.91 ± 4.56 | P=0.666 |

@: Only males were tested in this experiment.

§: Olfactory cues indicate the volatile organic compounds of branches of *A. negundo* with green leaves.

Statistical analysis: t indicates a two-tailed independent-samples Student t-test, MW indicates a Mann-Whitney test.
