# Supplementary material for: Integration of Visual and Olfactory Cues in Host Plant Identification by the Asian Longhorned Beetle, Anoplophora glabripennis (Motschulsky) (Coleoptera: Cerambycidae)
Source: PLoS One. 2015 Nov 10;10(11):e0142752. doi: 10.1371/journal.pone.0142752 (PMC4640517; doi:10.1371/journal.pone.0142752)
Supplement: S3 Table — (DOC) [file pone.0142752.s003.doc]

**S 3 table. Relevant data underlying the findings described in manuscript**

**Experiment 1: visual and/or olfactory cues of host versus non-host plants**

***1. Number of first orientating and first visits for each cue of host plants (A. negundo) and non-host plants (S. chinensis and P. bungeana) for A. glabripennis***

| Experiment | Type of cue | Options offered to ALB | First orientating |  |  | First visits |  |  |
| --- | --- | --- | --- | --- | --- | --- | --- | --- |
|  |  |  | Female | Male | No. of non-responses | Female | Male | No. of non-responses |
| 1.1 | Visual cues | *A. negundo* | 15 | 11 | Female: 0 | 14 | 12 | Female: 4 |
|  |  | *S. chinensis* | 6 | 5 | Male: 0 | 3 | 2 | Male: 2 |
|  | Olfactory cues | *A. negundo* | 18 | 10 | Female: 0 | 17 | 13 | Female: 1 |
|  |  | *S. chinensis* | 4 | 7 | Male: 0 | 4 | 2 | Male: 2 |
|  | Visual +olfactory cues | *A. negundo* | 14 | 13 | Female: 0 | 15 | 16 | Female: 2 |
|  |  | *S. chinensis* | 7 | 6 | Male: 0 | 4 | 2 | Male: 1 |
| 1.2 | Visual cues | *A. negundo* | 13 | 13 | Female: 0 | 16 | 16 | Female: 1 |
|  |  | *P. bungeana* | 9 | 6 | Male: 0 | 5 | 2 | Male: 1 |
|  | Olfactory cues | *A. negundo* | 12 | 12 | Female: 0 | 12 | 11 | Female: 3 |
|  |  | *P. bungeana* | 8 | 8 | Male: 0 | 5 | 5 | Male: 4 |
|  | Visual +olfactory cues | *A. negundo* | 15 | 14 | Female: 0 | 17 | 16 | Female: 1 |
|  |  | *P. bungeana* | 7 | 6 | Male: 0 | 4 | 2 | Male: 2 |

**2. Data of latency and permanence times (in seconds) of *A. glabripennis* in response to each cue of host plants (*A. negundo*) and non-host plants (*S. chinensis* and *P. bungeana*)**

2.1 *A. negundo* vs. *P. bungeana*

Latency

|  | Visual cues |  |  |  | Olfactory cues |  |  |  | Vsiaul+olfactory cues |  |  |  |
| --- | --- | --- | --- | --- | --- | --- | --- | --- | --- | --- | --- | --- |
|  | *A. negundo* |  | *P. bungeana* |  | *A. negundo* |  | *P. bungeana* |  | *A. negundo* |  | *P. bungeana* |  |
|  | Female | Male | Female | Male | Female | Male | Female | Male | Female | Male | Female | Male |
| 1 | 36 | 215 | 108 | 51 | 123 | 183 | 95 | 180 | 164 | 55 | 65 | 208 |
| 2 | 58 | 205 | 219 | 162 | 285 | 85 | 52 | 149 | 244 | 245 | 107 | 24 |
| 3 | 267 | 96 | 47 |  | 95 | 51 | 541 | 138 | 167 | 400 | 47 |  |
| 4 | 423 | 253 | 84 |  | 34 | 260 | 157 | 201 | 151 | 90 | 145 |  |
| 5 | 72 | 24 | 82 |  | 161 | 318 | 77 | 194 | 222 | 125 |  |  |
| 6 | 90 | 92 |  |  | 142 | 276 |  |  | 220 | 293 |  |  |
| 7 | 260 | 458 |  |  | 380 | 327 |  |  | 209 | 215 |  |  |
| 8 | 60 | 50 |  |  | 125 | 250 |  |  | 223 | 71 |  |  |
| 9 | 108 | 90 |  |  | 151 | 250 |  |  | 141 | 64 |  |  |
| 10 | 410 | 141 |  |  | 149 | 252 |  |  | 38 | 165 |  |  |
| 11 | 219 | 187 |  |  | 142 | 301 |  |  | 61 | 230 |  |  |
| 12 | 119 | 214 |  |  | 202 |  |  |  | 100 | 53 |  |  |
| 13 | 22 | 327 |  |  |  |  |  |  | 106 | 200 |  |  |
| 14 | 92 | 110 |  |  |  |  |  |  | 155 | 148 |  |  |
| 15 | 69 | 133 |  |  |  |  |  |  | 137 | 98 |  |  |
| 16 | 212 | 30 |  |  |  |  |  |  | 57 | 160 |  |  |
| 17 |  |  |  |  |  |  |  |  | 182 |  |  |  |

Permanence

|  | Visual cues |  |  |  | Olfactory cues |  |  |  | Vsiaul+olfactory cues |  |  |  |
| --- | --- | --- | --- | --- | --- | --- | --- | --- | --- | --- | --- | --- |
|  | *A. negundo* |  | *P. bungeana* |  | *A. negundo* | Male | *P. bungeana* |  | *A. negundo* |  | *P. bungeana* |  |
|  | Female | Male | Female | Male | Female |  | Female | Male | Female | Male | Female | Male |
| 1 | 15 | 17 | 47 | 13 | 16 | 15 | 50 | 58 | 20 | 112 | 18 | 16 |
| 2 | 64 | 23 | 36 | 44 | 24 | 24 | 11 | 21 | 20 | 16 | 53 | 12 |
| 3 | 53 | 30 | 15 |  | 14 | 33 | 20 | 18 | 95 | 70 | 18 |  |
| 4 | 18 | 55 | 15 |  | 33 | 35 | 13 | 21 | 11 | 55 | 19 |  |
| 5 | 21 | 60 | 13 |  | 24 | 13 | 60 | 11 | 16 | 155 |  |  |
| 6 | 88 | 15 |  |  | 33 | 13 |  |  | 19 | 17 |  |  |
| 7 | 14 | 46 |  |  | 16 | 60 |  |  | 20 | 40 |  |  |
| 8 | 25 | 58 |  |  | 18 | 60 |  |  | 20 | 13 |  |  |
| 9 | 20 | 21 |  |  | 86 | 71 |  |  | 15 | 23 |  |  |
| 10 | 26 | 30 |  |  | 24 | 117 |  |  | 26 | 55 |  |  |
| 11 | 34 | 62 |  |  | 37 | 20 |  |  | 17 | 24 |  |  |
| 12 | 23 | 33 |  |  | 34 |  |  |  | 32 | 41 |  |  |
| 13 | 40 | 20 |  |  |  |  |  |  | 21 | 39 |  |  |
| 14 | 17 | 15 |  |  |  |  |  |  | 22 | 80 |  |  |
| 15 | 30 | 47 |  |  |  |  |  |  | 22 | 12 |  |  |
| 16 | 70 | 27 |  |  |  |  |  |  | 25 | 26 |  |  |
| 17 |  |  |  |  |  |  |  |  | 14 |  |  |  |

2.2 *A. negundo* vs. *S. chinensis*

Latency

|  | Visual cues |  |  |  | Olfactory cues |  |  |  | Vsiaul+olfactory cues |  |  |  |
| --- | --- | --- | --- | --- | --- | --- | --- | --- | --- | --- | --- | --- |
|  | *A. negundo* |  | *S. chinensis* |  | *A. negundo* |  | *S. chinensis* |  | *A. negundo* |  | *S. chinensis* |  |
|  | Female | Male | Female | Male | Female | Male | Female | Male | Female | Male | Female | Male |
| 1 | 171 | 235 | 36 | 255 | 68 | 382 | 40 | 48 | 55 | 200 | 62 | 210 |
| 2 | 61 | 281 | 224 | 92 | 98 | 101 | 290 | 365 | 139 | 160 | 65 | 155 |
| 3 | 340 | 101 | 239 |  | 51 | 189 | 120 |  | 69 | 88 | 135 |  |
| 4 | 181 | 179 |  |  | 348 | 233 | 176 |  | 59 | 160 | 175 |  |
| 5 | 181 | 225 |  |  | 130 | 212 |  |  | 142 | 64 |  |  |
| 6 | 49 | 264 |  |  | 63 | 52 |  |  | 50 | 50 |  |  |
| 7 | 240 | 192 |  |  | 86 | 336 |  |  | 75 | 148 |  |  |
| 8 | 289 | 81 |  |  | 357 | 130 |  |  | 100 | 72 |  |  |
| 9 | 388 | 255 |  |  | 95 | 117 |  |  | 250 | 115 |  |  |
| 10 | 82 | 170 |  |  | 95 | 107 |  |  | 520 | 222 |  |  |
| 11 | 146 | 247 |  |  | 61 | 186 |  |  | 150 | 209 |  |  |
| 12 | 57 | 85 |  |  | 257 | 67 |  |  | 127 | 100 |  |  |
| 13 | 42 |  |  |  | 240 | 410 |  |  | 81 | 209 |  |  |
| 14 | 161 |  |  |  | 11 |  |  |  | 185 | 101 |  |  |
| 15 |  |  |  |  | 131 |  |  |  | 90 | 155 |  |  |
| 16 |  |  |  |  | 90 |  |  |  |  | 167 |  |  |
| 17 |  |  |  |  | 75 |  |  |  |  |  |  |  |

Permanence

|  | Visual cues |  |  |  | Olfactory cues |  |  |  | Vsiaul+olfactory cues |  |  |  |
| --- | --- | --- | --- | --- | --- | --- | --- | --- | --- | --- | --- | --- |
|  | *A. negundo* |  | *S. chinensis* |  | *A. negundo* |  | *S. chinensis* |  | *A. negundo* |  | *S. chinensis* |  |
|  | Female | Male | Female | Male | Female | Male | Female | Male | Female | Male | Female | Male |
| 1 | 19 | 25 | 19 | 30 | 55 | 56 | 51 | 16 | 30 | 35 | 105 | 31 |
| 2 | 14 | 38 | 11 | 21 | 42 | 34 | 26 | 18 | 41 | 70 | 19 | 79 |
| 3 | 30 | 25 | 35 |  | 22 | 40 | 29 |  | 20 | 22 | 20 |  |
| 4 | 100 | 28 |  |  | 49 | 21 | 19 |  | 30 | 36 | 20 |  |
| 5 | 23 | 14 |  |  | 60 | 27 |  |  | 181 | 33 |  |  |
| 6 | 38 | 63 |  |  | 60 | 40 |  |  | 53 | 100 |  |  |
| 7 | 48 | 73 |  |  | 29 | 11 |  |  | 25 | 90 |  |  |
| 8 | 12 | 48 |  |  | 55 | 18 |  |  | 24 | 13 |  |  |
| 9 | 18 | 50 |  |  | 90 | 40 |  |  | 25 | 100 |  |  |
| 10 | 46 | 45 |  |  | 17 | 33 |  |  | 18 | 17 |  |  |
| 11 | 28 | 62 |  |  | 39 | 60 |  |  | 26 | 34 |  |  |
| 12 | 26 | 90 |  |  | 22 | 14 |  |  | 22 | 42 |  |  |
| 13 | 18 |  |  |  | 17 | 42 |  |  | 51 | 30 |  |  |
| 14 | 32 |  |  |  | 17 |  |  |  | 47 | 27 |  |  |
| 15 |  |  |  |  | 50 |  |  |  | 117 | 14 |  |  |
| 16 |  |  |  |  | 70 |  |  |  |  | 106 |  |  |
| 17 |  |  |  |  | 44 |  |  |  |  |  |  |  |

**Experiment 2: the relative importance of visual or olfactory cues of host plants**

**1. Number of first orientating and first visits for each cue of host plants (*A. negundo*), black paper and blank control for *A. glabripennis***

| Experiment | Type of cue | Options offered to ALB | First orientating |  |  | First visits |  |  |
| --- | --- | --- | --- | --- | --- | --- | --- | --- |
|  |  |  | Female | Male | No. of non-responses | Female | Male | No. of non-responses |
| 2.0 | Visual cues | Black paper | 13 | 14 | Female: 0 | 10 | 10 | Female: 4 |
|  |  | Blank control | 15 | 11 | Male: 0 | 14 | 12 | Male: 3 |
|  |  | White paper@ |  | 15 |  |  | 17 |  |
|  |  | Blank control |  | 6 | Male: 0 |  | 3 | Male: 1 |
|  | Visual + olfactory cues | Black paper + olfactory cues^§^ | 11 | 10 | Female: 0 | 10 | 9 | Female: 1 |
|  |  | Blank control + olfactory cues^§^ | 9 | 10 | Male: 0 | 9 | 9 | Male: 2 |
| 2.1 | Visual cues | *A. negundo* | 13 | 11 | Female: 0 | 15 | 11 | Female: 1 |
|  |  | Blank control | 7 | 5 | Male: 0 | 4 | 4 | Male: 1 |
|  | Olfactory cues | *A. negundo* | 14 | 13 | Female: 0 | 15 | 14 | Female: 2 |
|  |  | Blank control | 7 | 7 | Male: 0 | 4 | 4 | Male: 2 |
|  | Visual +olfactory cues | *A. negundo* | 13 | 12 | Female: 0 | 15 | 14 | Female: 3 |
|  |  | Blank control | 6 | 5 | Male: 0 | 2 | 3 | Male: 0 |
| 2.2 | *A. negundo* | Visual cues | 17 | 21 | Female: 0 | 15 | 23 | Female: 1 |
|  |  | Olfactory cues | 6 | 15 | Male: 3 | 7 | 11 | Male: 5 |
|  | *A. negundo* | Visual cues | 11 | 10 | Female: 0 | 7 | 6 | Female: 2 |
|  |  | Visual +olfactory cues | 13 | 14 | Male: 0 | 15 | 14 | Male: 4 |
|  | *A. negundo* | Olfactory cues | 7 | 4 | Female: 0 | 3 | 2 | Female: 3 |
|  |  | Visual +olfactory cues | 15 | 18 | Male: 0 | 16 | 18 | Male: 2 |

**2. Data of latency and permanence times (in seconds) of *A. glabripennis* in response to each cue of host plants (*A. negundo*), black paper and blank control**

2.1 Latency

|  | **Exp.2.0** |  |  |  |  |  |  |  |  |  | **Exp.2.1** Visual cues | | | | Olfactory cues | | | |
| --- | --- | --- | --- | --- | --- | --- | --- | --- | --- | --- | --- | --- | --- | --- | --- | --- | --- | --- |
|  | Black paper | | Blank control | | White paper | Blank control | Black paper + olfactory cues | | Blank control + olfactory cues | | *A. negundo* | | Blank control | | *A. negundo* | | Blank control | |
|  | Female | Male | Female | Male | Male | Male | Female | Male | Female | Male | Female | Male | Female | Male | Female | Male | Female | Male |
| 1 | 267 | 503 | 57 | 285 | 240 | 500 | 211 | 180 | 231 | 68 | 157 | 66 | 370 | 239 | 67 | 172 | 145 | 234 |
| 2 | 34 | 207 | 34 | 423 | 551 | 150 | 200 | 148 | 133 | 158 | 203 | 61 | 370 | 195 | 490 | 128 | 49 | 53 |
| 3 | 320 | 85 | 123 | 108 | 300 | 30 | 65 | 139 | 141 | 258 | 137 | 125 | 250 | 88 | 10 | 344 | 102 | 45 |
| 4 | 353 | 347 | 315 | 261 | 51 |  | 65 | 111 | 250 | 195 | 183 | 71 | 150 | 66 | 150 | 245 | 397 | 42 |
| 5 | 146 | 51 | 116 | 263 | 212 |  | 92 | 195 | 375 | 78 | 64 | 121 |  |  | 71 | 214 |  |  |
| 6 | 147 | 170 | 64 | 380 | 99 |  | 126 | 201 | 321 | 395 | 152 | 54 |  |  | 230 | 180 |  |  |
| 7 | 458 | 137 | 109 | 179 | 81 |  | 425 | 221 | 72 | 181 | 44 | 290 |  |  | 77 | 430 |  |  |
| 8 | 38 | 185 | 210 | 320 | 285 |  | 85 | 207 | 78 | 128 | 145 | 361 |  |  | 54 | 12 |  |  |
| 9 | 187 | 72 | 152 | 396 | 443 |  | 118 | 241 | 191 | 168 | 150 | 125 |  |  | 73 | 78 |  |  |
| 10 | 253 | 148 | 36 | 379 | 110 |  | 195 |  |  |  | 319 | 85 |  |  | 400 | 180 |  |  |
| 11 |  |  | 262 | 200 | 153 |  |  |  |  |  | 142 | 345 |  |  | 52 | 172 |  |  |
| 12 |  |  | 64 | 339 | 139 |  |  |  |  |  | 31 |  |  |  | 224 | 140 |  |  |
| 13 |  |  | 185 |  | 112 |  |  |  |  |  | 359 |  |  |  | 127 | 93 |  |  |
| 14 |  |  | 276 |  | 76 |  |  |  |  |  | 200 |  |  |  | 140 | 75 |  |  |
| 15 |  |  |  |  | 114 |  |  |  |  |  | 111 |  |  |  | 213 |  |  |  |
| 16 |  |  |  |  | 40 |  |  |  |  |  |  |  |  |  |  |  |  |  |
| 17 |  |  |  |  | 104 |  |  |  |  |  |  |  |  |  |  |  |  |  |

|  | Visual + olfactory cue | | | | **Exp. 2.2** *A. negundo* | | | | *A. negundo* | | | | *A. negundo* | | | |
| --- | --- | --- | --- | --- | --- | --- | --- | --- | --- | --- | --- | --- | --- | --- | --- | --- |
|  | *A. negundo* | | Blank control | | Visual cues | | Olfactory cues | | Visual cues | | Visual + olfactory cue | | Olfactory cues | | Visual + olfactory cue | |
|  | Female | Male | Female | Male | Female | Male | Female | Male | Female | Male | Female | Male | Female | Male | Female | Male |
| 1 | 178 | 204 | 74 | 100 | 8 | 109 | 95 | 66 | 75 | 62 | 258 | 130 | 342 | 102 | 15 | 181 |
| 2 | 210 | 210 | 461 | 80 | 18 | 40 | 214 | 101 | 285 | 62 | 455 | 225 | 14 | 90 | 228 | 42 |
| 3 | 272 | 147 |  | 103 | 138 | 109 | 329 | 49 | 74 | 26 | 146 | 267 | 310 |  | 49 | 198 |
| 4 | 44 | 345 |  |  | 405 | 80 | 167 | 148 | 205 | 65 | 120 | 11 |  |  | 200 | 385 |
| 5 | 349 | 193 |  |  | 371 | 71 | 466 | 141 | 361 | 173 | 300 | 164 |  |  | 238 | 200 |
| 6 | 81 | 141 |  |  | 90 | 214 | 130 | 75 | 310 | 262 | 67 | 21 |  |  | 218 | 138 |
| 7 | 438 | 76 |  |  | 184 | 34 | 169 | 199 | 52 |  | 142 | 30 |  |  | 85 | 65 |
| 8 | 305 | 262 |  |  | 280 | 198 |  | 58 |  |  | 90 | 143 |  |  | 410 | 90 |
| 9 | 110 | 413 |  |  | 380 | 105 |  | 95 |  |  | 330 | 134 |  |  | 79 | 292 |
| 10 | 70 | 238 |  |  | 120 | 60 |  | 146 |  |  | 79 | 88 |  |  | 99 | 201 |
| 11 | 207 | 339 |  |  | 320 | 245 |  | 22 |  |  | 87 | 108 |  |  | 51 | 150 |
| 12 | 128 | 122 |  |  | 60 | 185 |  |  |  |  | 408 | 92 |  |  | 291 | 20 |
| 13 | 309 | 122 |  |  | 255 | 72 |  |  |  |  | 314 | 67 |  |  | 90 | 18 |
| 14 | 199 | 77 |  |  | 193 | 85 |  |  |  |  | 223 | 46 |  |  | 212 | 113 |
| 15 | 83 |  |  |  | 105 | 91 |  |  |  |  | 118 |  |  |  | 340 | 170 |
| 16 |  |  |  |  |  | 130 |  |  |  |  |  |  |  |  | 395 | 222 |
| 17 |  |  |  |  |  | 37 |  |  |  |  |  |  |  |  |  | 101 |
| 18 |  |  |  |  |  | 19 |  |  |  |  |  |  |  |  |  | 320 |
| 19 |  |  |  |  |  | 122 |  |  |  |  |  |  |  |  |  |  |
| 20 |  |  |  |  |  | 220 |  |  |  |  |  |  |  |  |  |  |
| 21 |  |  |  |  |  | 75 |  |  |  |  |  |  |  |  |  |  |
| 22 |  |  |  |  |  | 265 |  |  |  |  |  |  |  |  |  |  |
| 23 |  |  |  |  |  | 220 |  |  |  |  |  |  |  |  |  |  |

2.2 Permanence

|  | **Exp.2.0** |  |  |  |  |  |  |  |  |  | **Exp.2.1** Visual cues | | | | Olfactory cues | | | |
| --- | --- | --- | --- | --- | --- | --- | --- | --- | --- | --- | --- | --- | --- | --- | --- | --- | --- | --- |
|  | Black paper | | Blank control | | White paper | Blank control | Black paper + olfactory cues | | Blank control + olfactory cues | | *A. negundo* | | Blank control | | *A. negundo* | | Blank control | |
|  | Female | Male | Female | Male | Male | Male | Female | Male | Female | Male | Female | Male | Female | Male | Female | Male | Female | Male |
| 1 | 16 | 13 | 22 | 35 | 19 | 14 | 21 | 45 | 27 | 124 | 23 | 115 | 40 | 29 | 35 | 40 | 17 | 14 |
| 2 | 16 | 15 | 22 | 17 | 29 | 25 | 25 | 17 | 39 | 30 | 31 | 17 | 11 | 12 | 12 | 16 | 22 | 26 |
| 3 | 20 | 37 | 20 | 35 | 50 | 15 | 24 | 17 | 49 | 30 | 16 | 31 | 15 | 17 | 12 | 23 | 20 | 65 |
| 4 | 17 | 16 | 57 | 16 | 29 |  | 22 | 17 | 15 | 35 | 16 | 12 | 60 | 12 | 24 | 60 | 20 | 19 |
| 5 | 71 | 28 | 31 | 44 | 42 |  | 33 | 40 | 30 | 50 | 16 | 15 |  |  | 30 | 56 |  |  |
| 6 | 23 | 61 | 10 | 15 | 51 |  | 13 | 35 | 12 | 25 | 15 | 46 |  |  | 47 | 49 |  |  |
| 7 | 62 | 14 | 43 | 36 | 14 |  | 30 | 17 | 22 | 40 | 65 | 22 |  |  | 106 | 18 |  |  |
| 8 | 28 | 31 | 17 | 30 | 40 |  | 20 | 21 | 11 | 20 | 36 | 52 |  |  | 17 | 53 |  |  |
| 9 | 13 | 16 | 31 | 104 | 45 |  | 21 | 70 | 24 | 30 | 29 | 68 |  |  | 155 | 30 |  |  |
| 10 | 12 | 16 | 14 | 32 | 40 |  | 50 |  |  |  | 45 | 13 |  |  | 11 | 277 |  |  |
| 11 |  |  | 14 | 30 | 14 |  |  |  |  |  | 33 | 14 |  |  | 23 | 16 |  |  |
| 12 |  |  | 15 | 52 | 14 |  |  |  |  |  | 33 |  |  |  | 66 | 32 |  |  |
| 13 |  |  | 21 |  | 16 |  |  |  |  |  | 22 |  |  |  | 15 | 14 |  |  |
| 14 |  |  | 12 |  | 18 |  |  |  |  |  | 34 |  |  |  | 89 | 25 |  |  |
| 15 |  |  |  |  | 14 |  |  |  |  |  | 19 |  |  |  | 20 |  |  |  |
| 16 |  |  |  |  | 64 |  |  |  |  |  |  |  |  |  |  |  |  |  |
| 17 |  |  |  |  | 18 |  |  |  |  |  |  |  |  |  |  |  |  |  |

|  | Visual + olfactory cue | | | | **Exp.2.2** *A. negundo* | | | | *A. negundo* | | | | *A. negundo* | | | |
| --- | --- | --- | --- | --- | --- | --- | --- | --- | --- | --- | --- | --- | --- | --- | --- | --- |
|  | *A. negundo* | | Blank control | | Visual cues | | Olfactory cues | | Visual cues | | Visual + olfactory cue | | Olfactory cues | | Visual + olfactory cue | |
|  | Female | Male | Female | Male | Female | Male | Female | Male | Female | Male | Female | Male | Female | Male | Female | Male |
| 1 | 22 | 32 | 150 | 34 | 13 | 30 | 95 | 19 | 30 | 65 | 63 | 28 | 28 | 58 | 19 | 33 |
| 2 | 26 | 16 | 26 | 36 | 71 | 18 | 34 | 17 | 39 | 78 | 30 | 30 | 76 | 20 | 42 | 46 |
| 3 | 30 | 24 |  | 43 | 22 | 32 | 33 | 34 | 45 | 37 | 10 | 16 | 20 |  | 82 | 22 |
| 4 | 47 | 188 |  |  | 65 | 33 | 43 | 16 | 39 | 40 | 15 | 51 |  |  | 20 | 60 |
| 5 | 138 | 170 |  |  | 15 | 44 | 14 | 27 | 17 | 17 | 17 | 74 |  |  | 24 | 15 |
| 6 | 13 | 62 |  |  | 35 | 31 | 30 | 25 | 29 | 36 | 15 | 60 |  |  | 86 | 77 |
| 7 | 23 | 15 |  |  | 25 | 17 | 115 | 26 | 23 |  | 66 | 34 |  |  | 100 | 50 |
| 8 | 19 | 34 |  |  | 17 | 18 |  | 39 |  |  | 12 | 56 |  |  | 35 | 23 |
| 9 | 88 | 23 |  |  | 22 | 16 |  | 10 |  |  | 20 | 12 |  |  | 16 | 37 |
| 10 | 20 | 48 |  |  | 33 | 33 |  | 19 |  |  | 13 | 40 |  |  | 71 | 84 |
| 11 | 27 | 48 |  |  | 35 | 18 |  | 16 |  |  | 60 | 67 |  |  | 73 | 47 |
| 12 | 37 | 120 |  |  | 12 | 30 |  |  |  |  | 38 | 18 |  |  | 47 | 57 |
| 13 | 12 | 22 |  |  | 16 | 17 |  |  |  |  | 22 | 13 |  |  | 23 | 19 |
| 14 | 120 | 44 |  |  | 52 | 18 |  |  |  |  | 15 | 40 |  |  | 15 | 16 |
| 15 | 33 |  |  |  | 30 | 37 |  |  |  |  | 30 |  |  |  | 60 | 83 |
| 16 |  |  |  |  |  | 22 |  |  |  |  |  |  |  |  | 82 | 18 |
| 17 |  |  |  |  |  | 20 |  |  |  |  |  |  |  |  |  | 65 |
| 18 |  |  |  |  |  | 160 |  |  |  |  |  |  |  |  |  | 14 |
| 19 |  |  |  |  |  | 11 |  |  |  |  |  |  |  |  |  |  |
| 20 |  |  |  |  |  | 60 |  |  |  |  |  |  |  |  |  |  |
| 21 |  |  |  |  |  | 29 |  |  |  |  |  |  |  |  |  |  |
| 22 |  |  |  |  |  | 106 |  |  |  |  |  |  |  |  |  |  |
| 23 |  |  |  |  |  | 21 |  |  |  |  |  |  |  |  |  |  |
